# Supplementary material for: Investigation on the Enzymatic Profile of Mulberry Alkaloids by Enzymatic Study and Molecular Docking
Source: Molecules. 2019 May 8;24(9):1776. doi: 10.3390/molecules24091776 (PMC6539310; doi:10.3390/molecules24091776)
Supplement: Supplementary file 1 [file molecules-24-01776-s001.pdf]

molecules

MDPI

Article

Investigation on the enzymatic profile of mulberry alkaloids by enzymatic study and molecular docking

Zhihua Liu[1], Ying Yang[2], Wujun Dong[1], Quan Liu[3], Renyun Wang[1], Jianmei Pang[1], Xuejun Xia[1], Xiangyang Zhu[4], Shuainan Liu[3], Zhufang Shen[3], Zhiyan Xiao[2] and Yuling Liu[1,*]

[1] State Key Laboratory of Bioactive Substance and Function of Natural Medicines, Institute of Materia Medica, Chinese Academy of Medical Sciences & Peking Union Medical College; 1 Xiannongtan Street, Beijing 100050, China; liuzhihua0207@163.com (Z.L.); dwujun@vip.sina.com (W. D.); wry@imm.ac.cn (R.W.); xjxia@imm.ac.cn (X.X.); 1191125290@qq.com (J.P.); ylliu@imm.ac.cn (Y.L.);

[2] Beijing Key Laboratory of Active Substance Discovery and Drug ability Evaluation, Institute of Materia Medica, Chinese Academy of Medical Sciences and Peking Union Medical College, Beijing 100050, China; yangying@imm.ac.cn (Y.Y.); xiaoz@imm.ac.cn (Z.X.);

[3] Pharmacology and Natural Medicine Research Laboratory, Institute of Materia Medica, Chinese Academy of Medical Sciences & Peking Union Medical College; 1 Xiannongtan Street, Beijing 100050, China; popliu@imm.ac.cn (Q.L.); liusn@imm.ac.cn (S.L.);shenzhuf@imm.ac.cn (Z.S.);

[4] Beijing Wehand-Bio Pharmaceutical Company Limited; 30 Tianfu Street, Beijing 102600, China; zhuxiangyang68@163.com (X.Z.)

* Correspondence: ylliu@imm.ac.cn; Tel.: +86-10-6315-9373; Fax: +86-10-89285090

Table S1. The docking scores of the small compounds with HPA based on the consensus score function

Name	1-DNJ	DAB	FA	acarbose	miglitol
LigScore1	0.97	3.28	2.01	5.54	2.51
LigScore2	2.72	4.01	3.61	6.17	3.79
PLP1 (−)	-8.59	33.97	12.67	111.96	39.16
PLP2 (−)	0.44	26.86	14.08	90.63	29.9
Jain	-1.38	-0.50	0.64	1.97	-0.88
PMF (−)	87.00	95.57	81.95	255.24	108.46
PMF04 (−)	55.15	66.12	57.78	184.30	73.03
Surflex_score (−)	5.91	4.59	3.80	12.89	4.81
Glide_score (−)	4.21	4.01	4.12	7.81	3.96
D_score (−)	91.53	82.14	79.27	236.36	91.32
G_score (−)	166.82	114.49	103.48	373.11	209.9
Chemscore (−)	9.92	10.91	10.76	1.96	3.19

Table S2. The docking scores of the small compounds with NtSI based on the consensus score function

Name	1-DNJ	DAB	FA	acarbose	miglitol
LigScore1	4.07	4.14	1.27	4.22	4.35
LigScore2	3.46	3.92	3.57	4.20	3.89
PLP1 (−)	43.84	24.92	21.50	43.93	22.98
PLP2 (−)	54.69	22.91	29.88	47.90	28.36
Jain	2.12	-0.25	-0.46	0.92	0.67
PMF (−)	150.38	117.87	128.52	76.46	153.88

PMF04 (−)	99.88	95.81	88.76	74.83	124.19
Surflex_score (−)	5.63	6.68	5.21	4.51	3.74
Glide_score (−)	5.79	5.52	5.07	4.50	4.74
D_score (−)	124.47	126.81	102.47	137.78	186.63
G_score (−)	158.96	216.12	176.39	218.56	195.31
Chemscore (−)	25.41	22.73	25.57	13.36	17.56

Table S3. The docking scores of the small compounds with NtMGAM based on the consensus score function

Name	1-DNJ	DAB	FA	acarbose	miglitol
LigScore1	6.34	3.88	3.78	5.06	5.26
LigScore2	5.77	4.29	4.67	4.22	5.05
PLP1 (−)	87.36	43.05	59.42	76.55	73.77
PLP2 (−)	82.44	49.05	66.83	82.48	79.96
Jain	7.07	3.88	5.15	1.54	4.70
PMF (−)	216.77	122.69	133.49	189.19	181.70
PMF04 (−)	162.02	79.41	88.82	151.09	124.81
Surflex_score (−)	8.34	6.20	6.62	2.62	7.57
Glide_score (−)	6.83	5.03	5.92	3.44	6.34
D_score (−)	103.40	78.95	97.88	140.40	118.45
G_score (−)	159.21	130.84	132.87	165.96	188.16
Chemscore (−)	3.28	4.24	5.78	-3.84	1.29

Table S4. The docking scores of the small compounds with CtMGAM based on the consensus score function

Name	1-DNJ	DAB	FA	acarbose	miglitol
LigScore1	5.35	3.83	4.77	6.25	5.51
LigScore2	5.43	4.51	4.48	6.56	5.11
PLP1 (−)	69.08	44.11	43.58	103.28	65.07
PLP2 (−)	79.15	47.92	53.08	91.38	73.52
Jain	6.68	2.32	3.38	2.85	6.91
PMF (−)	155.25	124.66	129.64	270.66	167.16
PMF04 (−)	103.07	78.60	79.04	201.80	112.31
Surflex_score (−)	7.82	6.55	5.74	12.05	5.92
Glide_score (−)	6.53	5.61	5.60	7.31	6.09
D_score (−)	112.50	90.42	99.30	232.24	122.12
G_score (−)	146.63	116.41	216.10	351.48	201.89
Chemscore (−)	6.10	8.90	5.20	-3.20	3.24

© 2019 by the authors. Submitted for possible open access publication under the terms and conditions of the Creative Commons Attribution (CC BY) license (http://creativecommons.org/licenses/by/4.0/).
